# Larval food quantity affects development time, survival and adult biological traits that influence the vectorial capacity of *Anopheles darlingi* under laboratory conditions

**DOI:** 10.1186/1475-2875-11-261

**Published:** 2012-08-02

**Authors:** Maisa da-Silva Araújo, Luiz Herman S Gil, Alexandre de-Almeida e-Silva

**Affiliations:** 1 Laboratory of Entomology, Tropical Pathology Research Institute/Oswaldo Cruz Foundation, Porto Velho, Rondonia, Brazil; 2 Laboratory of Insect Bioecology, Department of Biology, Federal University of Rondonia, Porto Velho, Rondonia, Brazil

**Keywords:** *Anopheles darlingi*, *Plasmodium*, Larval development, Vectorial capacity, Rondonia

## Abstract

**Background:**

The incidence of malaria in the Amazon is seasonal and mosquito vectorial capacity parameters, including abundance and longevity, depend on quantitative and qualitative aspects of the larval diet. *Anopheles darlingi* is a major malaria vector in the Amazon, representing >95% of total *Anopheles* population present in the Porto Velho region. Despite its importance in the transmission of the *Plasmodium* parasite, knowledge of the larval biology and ecology is limited. Studies regarding aspects of adult population ecology are more common than studies on larval ecology. However, in order develop effective control strategies and laboratory breeding conditions for this species, more data on the factors affecting vector biology is needed. The aim of the present study is to assess the effects of larval food quantity on the vectorial capacity of *An. darling* under laboratory conditions.

**Methods:**

*Anopheles darlingi* was maintained at 28°C, 80% humidity and exposed to a daily photoperiod of 12 h. Larvae were divided into three experimental groups that were fed either a low, medium, or high food supply (based on the food amounts consumed by other species of culicids). Each experiment was replicated for six times. A cohort of adults were also exposed to each type of diet and assessed for several biological characteristics (e.g. longevity, bite frequency and survivorship), which were used to estimate the vectorial capacity of each experimental group.

**Results:**

The group supplied with higher food amounts observed a reduction in development time while larval survival increased. In addition to enhanced longevity, increasing larval food quantity was positively correlated with increasing frequency of bites, longer blood meal duration and wing length, resulting in greater vectorial capacity. However, females had greater longevity than males despite having smaller wings.

**Conclusions:**

Overall, several larval and adult biological traits were significantly affected by larval food availability. Greater larval food supply led to enhance larval and production and larger mosquitoes with longer longevity and higher biting frequency. Thus, larval food availability can alter important biological traits that influence the vectorial capacity of *An. darlingi*.

## Background

Mosquitoes (Diptera: Culicidae) are medically the most important group of insects due to the disease they transmit and the magnitude of health problems that these diseases cause worldwide. The important role of vectors in the transmission of malaria, yellow fever, dengue fever and filariasis have led to intensive studies of their biology. Since the beginning of the 20th century, considerable attention has been given to the food requirements of the larvae, in order to aim to reduce or eliminate food supplies in larval natural breeding sites [1].

Both quantitative and qualitative aspects of larval nutrition are important to mosquito development and survival [2], and also to the emergence of adults [3]. Several species of mosquito larvae are non-selective filter feeders of organic particles suspended in water and of micro-organisms such as bacteria, viruses, protozoans and fungi [4]. Furthermore, pollen [5] as well as algae and bacteria [2] contribute to their development. Bacteria are the most abundant micro-organisms present in larval food, and mosquito growth can occur with bacteria as the only food source [6]. Conversely, adults, require protein for their development, mainly for pupation [7].

Currently, larval feeding studies aim to provide information to laboratory breeding; studies for potential larvicides, since there is no qualitative selectivity of particles ingested [4], and also basic biological knowledge on the effect of quality and quantity of food intake on growth, development and reproduction of insects in general [8].

Previous studies have shown that food availability affects larval development [6,9,10], adult emergence, sexual maturation, fecundity, survival [11], body size [6,12] and nutrient reserves [13].

Several studies investigated the relationship between food quantity, larval development and adult production. Wallace and Merritt [14] performed laboratory experiments with *Anopheles quadrimaculatus* and argued that larval survival was greater with enhanced food resources. *Anopheles maculipennis* larvae fed improperly displayed delayed larval development and decreased survival to the fourth instar [1].

According to Nelson [15], the body size of various species of culicides species has a genetic basis, but is also influenced by environmental factors. An increase in body size increases the probability of survival and success in the acquiring blood meal and in some species, the parasite infectivity [16], the parity and the vectorial capacity (VC) [17].

The VC, ie, the property of a vector to transmit the pathogen resulting in new cases of a specific disease, has increasingly attracted attention from researchers interested in its relationship with larvae food availability because it affects adult size and survival [14].

Tun-Lin *et al.*[9] related that increased size in *Aedes aegypti* led to higher VC and Schneider *et al.*[18] argued that the adult mosquito size of *Culex* might be a determinant factor for its VC.

The incidence of malaria in Rondonia, as in other tropical regions, is seasonal and related to variation in the number of breeding sites as well as the physico-chemical characteristics of breeding sites [19], which can alter the food availability for vector in the immature stages, and thus affecting the biological parameters previously mentioned.

*Anopheles darlingi* is the main malaria vector in the Amazon, accounting for 99% of the mosquitoes captured in the Rondonia region [20]. Despite the importance of *An. darlingi* in disease transmission, knowledge about the biology and ecology of the larval form is limited.

The larval biology studies have evaluated factors that affect the biology vector, such as longevity, VC, adult size and dispersal [14]. Knowledge of larval biology could contribute to modelling of population dynamic [21] and improving vector control measures [22,23]. In addition, it may improve existing *An. darlingi* rearing procedures in the laboratory in order to promote faster larval development, higher survival rates, and production of homogeneous adult population. The goal of this study is to investigate the effects of the larval food amount on the biology (eg, larval development, survival, adult survival and size) of the vector *An. darlingi* and its impact on the VC under laboratory conditions.

## Methods

### Rearing *Anopheles darlingi*

Mosquitoes were collected using protected human landing catches performed by adult volunteers (authors of the present study) from 18:00 and 21:00 h at Vila Candelária in Porto Velho, Rondonia. Ethic clearance of the procedure was given by the Ethic Committee of the CEPEM (n° 056/2007) which included written and signed information about the risk of acquiring malaria during captures. An average of one hundred mosquitoes were blood-fed on rabbits for 15 min the next day after field catches. Females were induced to oviposition, removing one of the wings using tweezers under a stereomicroscope and the placed individually in small plastic cups filled distilled water [24]. The next morning, females with more than one hundred eggs were used in the experiments and replicates comprised of 100 eggs from a single female.

Mosquitoes were reared in insect-rearing chambers maintained at 27 ± 1°C and 80% relative humidity and exposed to fluorescent light for 12 h daily combined with daylight [7]. Early hatched first instar larvae were kept in plastic pans (40 × 40 × 5 cm) containing 1 l of distilled water and 100 larvae/pan and fed with finely grinded fish food (TetraMin Tropical Flakes-Spectrum Brands, Inc). The rearing pans were inspected daily to maintain consistent water levels and remove debris from the water using plastic pipettes whenever necessary. Pupae were transferred to disposable plastic cups containing distilled water and stored in empty cages until adult emergence.

The first generation (F_1_) larvae were fed with different food amounts (Table 1) and some biological traits of larvae and adult were analysed.

**Table 1:** **Food amounts (mg) supplied to the larvae of *Anopheles darlingi ***

**Instars**	**Food amount**		
	**Low **	**Medium **	**High **
1	0.18	0.35	0.6
2	2.5	5	10
3	5	10	15
4	10	15	30

The amount corresponds to 100 larvae. In the first and second instars, the food is offered only once a day, for the third instar twice and to the third and fourth instars, three times a day.

Food supply based on the food amounts consumed by other culicid species [7].

### Effects of food amount on biology of *Anopheles darlingi*

Batches of larvae were divided into three experimental groups: low, medium and high food supply based on the food amounts consumed by other species of culicids [7]. There were six replicates for each treatment. Larval development and survival was recorded twice daily (06:00 and 18:00). Larval exuviae and dead individuals were removed and counted. Mortality at each stage and the number of emerged adult males and females were recorded. Larvae were counted daily and categorized according to instars as determined visually. The presence of exuviae indicated changes of larval instars [25].

### Duration of larval development, mortality rate and longevity of adults

The basic methodology developed by Forattini *et al.*[26] for calculating of average duration of developmental stages by means of the variable median stage of insects was applied here to evaluate mosquito stage duration. The median stage of raising (E_*i*_) in each inspection was noted in intervals of 12 h, to determine the median stage of distribution of frequencies of the number of individuals in the various stages. The numbers 1 to 4 correspond to the first, second, third, fourth larval instars, respectively, 5 to the stage of pupa and 6 to the adult. Survival rate of each stage (sj), survival rate (S) and mortality rate (IM) were calculated according to Bergo *et al.*[25]:

Survival rate of each stage (sj)

(1) $$\text{sj}=\frac{1-\text{ dead larvae at stage j}}{\;\text{n}{}^{\circ}\;\text{survivors at stage j}}$$

Survival rate total (S)

(2) $$\left(\text{S}\right)={\text{ s}}_{1+}{\text{s}}_{2+....+}{\text{s}}_{5}$$

S_1_: n° of larvae in the beginning of stage 1.

S_2_: n° of larvae in the beginning of stage 5.

The number of days that adult (five individuals) survived, ie, not inseminated females and males of each treatment (six replicates), was used to determine adult longevity. These mosquitoes were maintained with 20% sucrose, water and periodic blood meals from a human arm twice a week for 15 min.

### Vectorial capacity

Vectorial capacity (VC) was calculated as [27]:

(3) $$\text{VC}=\frac{{\text{ma}}^{2}{\text{p}}^{\text{n}}}{-{\text{log}}_{\text{e}}\text{p}}$$

Vector density (m) was held constant (5 females), survivorship (p) and average bite frequency (a) were determined in the laboratory [28]. Daily survival rate (p) was calculated by the formula p = ^d^√P, in which d is duration of the study (10 days) and P the proportion of females survive by the end of that period [29]. Five newly emerged females from each experimental group were placed individually in plastic cages and offered a blood meal daily during 10 minutes in the arms of a human host (author of the present work). The proportion of females that ingested or attempted to ingest blood from the human host was used to estimate bite frequency [28]. The extrinsic incubation period (n) in the mosquito for *Plasmodium falciparum* used was 16 days at the 27°C, according to Klein *et al.*[30].

### Wing length

One wing of each mosquito was mounted on a slide and measured using a binocular microscope equipped with an ocular micrometer (accuracy of 0.1 mm). The wing length was measured as the distance from axillary incision (alula) to the apical margin (radius veins) [31].

### Data analysis

The effect of food amount in the biology of the vector was (larval development, survival, adult longevity, biting frequency and wing length) was analysed by One Way ANOVA on ranks (Kruskal-Wallis). Correlation among different variables were performed in tests using Pearson for parametric data and Spearman for non-parametric data (SigmaStat 2.03 SPSS Inc., 1992–1997).

## Results and Discussion

**Figure 1:**
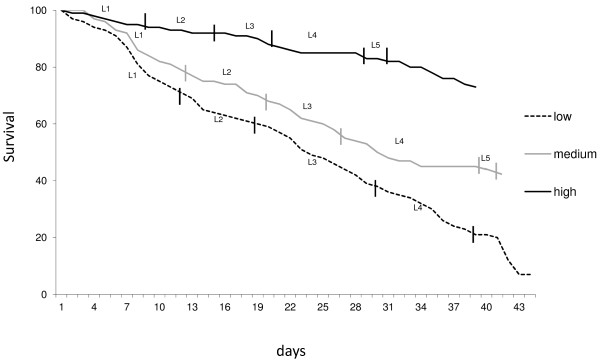
**Survival and average instar duration of *Anopheles darlingi *larvae fed with increasing food amounts.** L1, L2, L3 and L4 indicate the first, second, third and fourth larval instars, respectively and L5 indicates the pupal stage. See Table 1 for food amount supplied to each instar.

The larval development time was affected by the different amounts of food provided daily (F = 77.98; P < 0.001). Generally, the development time decreased with increasing food (Figure 1). This result agrees with similar studies of other mosquito species, eg, *Anopheles stephennsi *[32], *A. aegypti *[9,33], *Toxorhynchites splendens *[10], *Anopheles gambiae *[34] and *An. quadrimaculatus *[35] where food availability varied over time.

The group supplied with the highest food amount had accelerated development during the early instars while the fourth instar had longer duration, usually, the longest duration (H = 63.4; P < 0.001) (Table 2). Furthermore, the group supplied with the lower food amount experienced longer first and third instar. According to Lara *et al.*[36], poor diet causes an extended larval period and since immature spend 25% of their biomass on average [37] moulting, mosquito larvae must acquire enough food supply for ecdysis to avoid a high mortality rate [38].

**Table 2:** **Survival and larval instar duration of *Anopheles darlingi *(Diptera: Culicidae) larvae fed with increasing food concentrations under controlled conditions **

**Duration of instars (days)**				**Survival (%)**		
**Food amount**				**Food amount**		
**Instars**	**Low**	**Medium**	**High**	**Low**	**Medium**	**High**
L1	11.5a1	12a2	8.5a2	67.5a1	84.0a2	96.5a3
L2	7.2b1	7.5a1	6.0b1	55.0b1	68.5b2	94.5b3
L3	11.0b1	6.7a12	5.5b2	38.5c1	57.5c2	89.0c3
L4	8.5b1	13.0b2	8.7a3	21.5d1	42.0d2	77.0d3
L5	1.9c1	1.8c1	1.8c1	13.0e 1	33.0e2	52,5e3

One way Anova on ranks (Kruskal-Wallis) and Student-Newman-Keuls (comparisons); n=6; Different letters indicates significant differences (P<0,05) for the same column. Different numbers indicates significant differences (P<0.05) for the same line. L1 – L4 corresponds to the four larval instars and the pupal to L5; See Table 1 for food amounts supplied to each instar.

The data indicates that the first and four instars took longer to develop especially when immature were supplied with low and medium food amounts. Notably, the fourth instar had the longest duration compared to other instars, probably because it precedes the pupal stage which possess the greatest amount of nutrition reserves that are required for the transition to adulthood via moulting [4,25,39]. Telang *et al.*[38] reported that a critical larval mass for reaching pupal stage in *A. aegypti* depended upon both hormonal control and nourishment of the.

Larval survival was also affected by the different food supply (F = 129.89; P < 0.001), which observed an increase in numbers with increasing food amounts; differences among all larval instars statistically significant (H = 76.4; P < 0.001). Overall, the last two instars accounted for most of the deaths in groups provided with the low food amount (Table 2).

The pupae survival significantly increased with increasing food amounts, probably due to nutrition reserves rich in lipids [8,40] that accumulated during the larval stage [4,6]. This may account for the high mortality observed for pupae subjected to low food supply.

Although the development time to adulthood was not measured here, past studies have observed that the males generally emerge before females in nature and the laboratory [37,41,42]. Furthermore, larvae survival and development time had a negative correlation. Similar results were previously reported for *An. darlingi *[25], *An. stephensi *[32], *A. aegypti *[9], *T. Splendens *[10], *Anopheles arabiensis *[5,43], *An. gambiae *[34] and *An. quadrimaculatus *[35].

Larval development time in *An. darlingi* was much longer compared to other mosquito species. Under natural conditions, the duration of the entire development period generally ranges from 12 to 14 days [4], depending on the mosquito gender [43]. However, shorter development time is frequently observed in mosquitoes, eg, *An. gambiae*[44]that breed in unstable and transient sites [4], or when submitted to predation [35].

*Anopheles gambiae* larvae that were similarly reared at 28°C and supplied with food *ad libitum*, completed their development in 9.9 to 11 days [45]. However, *An. gambiae* mosquito development occurred more quickly to other anophelines species [46].

Santos *et al.*[47] studied the biology of *An. darlingi* under laboratory conditions and reported that the transition from egg to adult took an average of 15.6 days with a survival rate of 57%. Bergo *et al.*[25] reported a shorter development period of 13.9 days and an overall survival of 95%. However, the quality and quantity of larval food differed substantially from the present work. Moreover, rearing conditions differed and therefore, precluding suitable comparison of data.

The larval development time of *An. darlingi* occurs within 9.5 days in the field; shorter than *An. darlingi* larvae reared in the laboratory [48]. Besides extrinsic factors, one possible explanation for this discrepancy between the field and laboratory result may be due to the presence of open water habitats that provide significantly more micro-invertebrate dietary resources for *An. darlingi* larvae.

The daily survival (p) of female larvae reared with low, medium and high amounts of food was 0.2, 0.4 and 0.6, respectively. The lifetime biting frequencies (a) were 0.7, 0.8 and 0.9, respectively. Therefore, *P. falciparum* incubated for 16 days extrinsically under a constant temperature of 27°C and constant vector density of five females/person would experience a 30-fold increase in vectorial capacity compared to the low and high fed experimental groups under laboratory conditions (Table 3).

**Table 3:** **Biological parameters of *Anopheles darlingi *females emerged from larvae fed with increasing food amounts under controlled conditions (27 ± 1°C; 80% RU and 12h photoperiod)**

**Food amount**	**Biting frequency ratio NS**	**Blood meal duration (min) NS**	**Survival (%)**	**Vectorial Capacity**	**Wing length (mm)**	**Longevity (days)**
Low	0.7	3.0	20.0a	2.5a	1.5	11.0a
Medium	0.8	2.8	40.0a	2.7b	12	13.0ab
High	0.9	3.6	60.0b	2.8b	52	14.5b

One way Anova on ranks (Kruskal-Wallis) and Dunn's method (comparisons); n=25 (Low food concentration) and n=30(Medium and high food concentrations); Different letters indicate significant differences (P<0.05) in each column. NS= not significant (P>0.05). See table 1 for food amounts supplied to each instar. Biting frequency: ratio of mosquitoes biting from a sample of 5 females caged individually during 10 days for each experimental condition.

The group fed with the high food amount had better adult survival (H = 19.60; P < 0.001) and longer wing length (H = 24.95; P < 0.001) than adults that emerged from larvae reared with a low food amount (Table 3).

Grimstad and Walker [12] and Lyimo and Koella [16] also related similar results with the mosquitoes *Aedes triseriatus* and *An. gambiae* s.l., respectively.

High population density of *An. darlingi* in the field relate to transformations in larval habits [49] because environmental variations, such as food resources, directly affect larval development and adult production as related in this work. Nutrition reserves acquired during the immature stages play a role in the reproductive success of the adult [50,51]. Since *An. darlingi* does not mate under laboratory conditions, the reproduction parameters of this species could not be evaluated. However, the amount of food availability to the larvae of other mosquito species, including *A. aegypti* and *Ochlerotatus atropalpus*, affected the number of mature primary egg follicles [50,51] and the number of eggs oviposited by *An. stephensis*[32], which consequently altered the VC.

**Figure 2:**
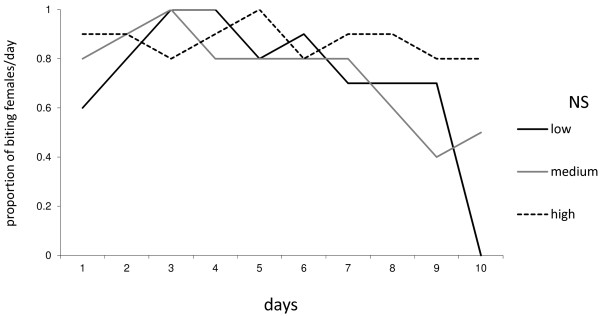
**Daily proportion of biting *Anopheles darlingi *emerged from larvae fed with increasing food amounts.** One Way Repeated measures ANOVA. NS: indicates no significant differences (P>0.05). See Table1 for food amount supplied to each instar.

Larvae supplied with the high food amount resulted in adults with higher longevity (H = 6.86; P = 0.03) (Table 3). In general, females survived better than males (H = 12.17; P < 0.001) (Figure 2A), but not when larvae were submitted to a lower food amount.

The longevity of *An. darlingi* adults was affected by food amount available during the larval stage. This was also reported for *An. stephensis*[32] and *An. arabiensis*[52]. Nutrient reserves of adults (mainly glycogen and triglycerides) obtained during the larval stage, contribute to an increase the longevity [7,37], which may explain the shorter longevity of adults derived from larvae supplied with low food amount.

*Anopheles darlingi* males had shorter lifespan than females. This was also related to other mosquito species [53,54] and the males were the first to emerge, with shorter time to obtain nutritional reserves. Moreover, females can increase their lifespan with multiple blood meals [28].

Female longevity varied from 11 to 15 days when larvae were subjected to increasing food amounts, which was lower compared to other tropical mosquito species with an average of 19 to 23 days [4]. However, this was sufficient time to allow the malaria parasite *P. falciparum* to complete development within *An. darlingi*[55].

Wing length was significantly affected by the food supply (H = 11.54; P < 0.001) with males possessing wings longer than females (Figure 2B).

Despite the significant margin, there was a positive correlation between wing size and the longevity of *An. darlingi*. Several studies explored the relation between vector size and longevity found that larger mosquitoes usually had higher survival rates than smaller ones [9,54,56], with a few exceptions [57].

Since *An. darlingi* females captured in the field displayed a wide-range of wing sizes compared with other *Anopheles* species, this suggested that these mosquitoes originated from different larval habitats [58]. Furthermore, the wing size of *An. darlingi* captured in peri-urban regions of Porto Velho, Rondonia varied significantly throughout the year (Batista, pers. comm.).

Time is important for males in seasonal breeding populations because males that emerge earlier can mate with more females. On the other hand, the effect of size on mating success is rather small. Body size is more important for females because it determines fecundity during lifetime [54]. In the present work, males were larger than females, contrary to the results from Lehmann *et al.*[54]. One possible explanation concerns larvae development since males that develop faster undergo ecdysis first. Thus, these males are favoured in the competition for food with females.

The mean wing size of *An. darlingi* females captured from the field was larger than individuals reared in laboratory [58]. Data obtained by Batista (pers. comm.) support these results for *An. darlingi*, but not for other species as related by Grieco *et al.*[48] and Lounibos *et al.*[58].

Body size of female mosquitoes affects dispersion, host attack rate [59], the number of eggs laid [56], and the success and frequency of blood meals [17]. The wing size of *An. darlingi* females that were fed high amounts as larvae was significantly larger than females that were fed low food amounts. The former females had higher biting frequency and a positive correlation between the wing size, biting frequency (R = 0.24; P = 0.02) and biting duration (R = 0.23; P = 0.03).

The relation between the body size and VC of mosquitoes has attracted the attention of researchers for a while. Smaller females often require two to three blood meals to develop their first batch of eggs. The frequency blood meals may increase the probability that smaller females acquire an infectious blood meal [2,18]. In addition, longer blood meals by larger females [18,32] may increase the number of gametocytes ingested and increasing the probability of infection by the vector [3]. The size of the mosquito also influences oocyst numbers in the midgut of naturally infected mosquitoes [16], supporting the hypothesis that mosquitoes arising from well nourished larvae are more competent for parasite transmission.

The biting frequency of *An. darlingi* over a 10-day period tends to decrease over time, differing from the results with *An. gambiae *[28]. However, field data indicated that the frequency of bites of *A. aegypti * decreases with age, leading to a decrease in VC [60].

**Figure 3:**
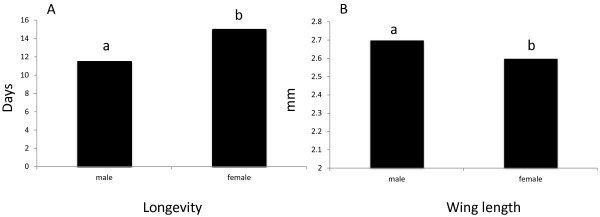
** Adult longevity (A) and wing length of male and female (B) *Anopheles darlingi *emerged from larvae fed with increasing food amounts.** Anova on ranks (Kruskal-Wallis) and Dunn's method (comparisons); n=38 (Low) and n=80 (Medium and High); Different letters indicate significant differences (P<0.05). See Table 1 for food amount supplied to each instar.

Females from high fed larvae had bite peaks close to three days, following the duration of gonotrophic cycle of *An. darlingi *[7], while those from low fed larvae had a consistent biting pattern, possibly to supply nutritional reserves that were not sufficient during the larval stage (Figure 3).

Wing size, adult longevity, and frequency of bites are important determinants of VC and fitness of mosquitoes. It is worth noting that larval development time also affects VC indirectly [61] as related in the present work. Finally, larval breeding environment changes, such as food resources, affects the population composition and adult fitness [18,62] and thus may affect the incidence of human malaria due to variations in the VC of *An. darlingi*.

## Conclusions

Multiple biological parameters of *An. darlingi* were significantly affected by the amount food provided to larvae. Larval development time was significantly longer and mortality rate was higher when larvae were fed with low food amounts. These conditions also resulted in a reduction in adult longevity and number of progeny, and a low VC (VC = 1.5). On the other hand, larvae fed with the high food amount produced adults with larger wing sizes and longer longevity, greatly increasing the VC (VC = 52).

Moreover, the data suggests rearing *An. darlingi* with high food quantity in laboratory, since a higher food supply accelerates larval development and improves adult fitness.

## Abbreviations

a: Average bit frequency; CEPEM: Research Center in Tropical Medicine; Ei: Median stage of raising; F1: First generation; IM: Mortality rate; m: Vector density; S: Survival rate; sj: Survival rate of each stage; p: Daily survival rate; VC: Vectorial capacity.

## Competing interests

The authors declare that they have no competing interests.

## Authors’ contributions

All authors contributed to the development of this work and provided comments on the manuscript. MSA performed the experiments supervised by AAS. MAS drafted the manuscript and AAS and LHSG reviewed manuscript. All authors read and approved the final manuscript.
